# Potential benefits from global warming to the thermal biology and locomotor performance of an endangered Patagonian lizard

**DOI:** 10.7717/peerj.7437

**Published:** 2019-08-09

**Authors:** Facundo Cabezas-Cartes, Jimena B. Fernández, Fernando Duran, Erika L. Kubisch

**Affiliations:** Laboratorio de Ecofisiología e Historia de vida de Reptiles, Instituto de Investigaciones en Biodiversidad y Medio Ambiente, Universidad Nacional del Comahue, CONICET, San Carlos de Bariloche, Río Negro, Argentina

**Keywords:** Climate change, Operative temperatures, Patagonia, Preferred temperatures, *Phymaturus*, Thermoregulation, Thermal performance curves, Vulnerability

## Abstract

Global warming can significantly affect many aspects of the biology of animal species, including their thermal physiology and physiological performance. Thermal performance curves provide a heuristic model to evaluate the impacts of temperature on the ecophysiology of ectotherms. When integrated with other thermal biology parameters, they can be used to predict the impacts of climate change on individual fitness and population viability. In this study, we combine holistic measures of thermal physiology and the thermal sensitivity of locomotor performance with environmental temperatures measured at fine scale to estimate the vulnerability to global warming of the endangered Patagonian lizard *Phymaturus tenebrosus*. Our results indicate that this lizard exhibits its preferred temperatures and maximum locomotor performance at higher temperatures than the mean temperature it currently experiences in its habitat. In addition, it exhibits a low effectiveness of thermoregulation, being a poor thermoregulator. In view of the results obtained, we suggest that the climatic conditions of Patagonia may be advantageous for *P. tenebrosus* to survive future global warming, since its thermal physiology and locomotor performance may improve under increasing in environmental temperatures in its habitat.

## Introduction

In recent decades, global warming has emerged as one of the most serious threats to biodiversity. In particular, it has been shown that rising temperatures can significantly affect many aspects of animal species’ biology, such as physiology (increased metabolic rates (Dillon, Wang & Huey, 2010)), morphology (shrinking body size (Sheridan & Bickford, 2011)), life cycle demography (Daufresne, Lengfellner & Sommer, 2009), life history (Bestion et al., 2015), phenology, and distribution (Parmesan & Yohe, 2003). As a consequence of these changes within species, community structure can also be affected (Walther, 2010). Understanding how climate change affects the physiology of different kinds of organisms is a critical challenge in modern ecology and evolutionary biology (Pörtner & Farrell, 2008). For example, the responses of endotherms are probably different from those of ectotherms (Bozinovic & Pörtner, 2015). Reptiles, as ectotherms, are particularly susceptible to changes in climate since most of their physiological functions, including locomotion, digestion, reproduction, and growth, are tightly correlated with environmental conditions, especially with temperature (Adolph & Porter, 1993; Angilletta, 2009). Moreover, in recent years several studies have reported current and predicted future potential extinctions of lizards’ populations as consequence of rising temperatures (Sinervo et al., 2010; Kubisch, Fernández & Ibargüengoytía, 2016; Vicenzi et al., 2017). However, other authors suggest that the effects of rising temperatures may be less detrimental than previously postulated, especially in species with high dispersal ability (Araújo, Thuiller & Pearson, 2006) or in species occurring at high latitudes or altitudes like some liolaemids (Bonino et al., 2015a; Gómez Alés et al., 2018).

The effects of increasing environmental temperatures on lizards’ populations will depend, at a fine scale, on the availability of microenvironmental temperatures that allow organisms to effectively thermoregulate and reach body temperatures (*T*_b_) within a range which allows peak performance of the most relevant physiological processes (Angilletta, Hill & Robson, 2002; Angilletta, 2009). However, this will also depend upon the effectiveness of thermoregulation of the species (*E*), which determines the degree to which thermoregulatory activity by the animals compensate for the thermal shortcomings of a particular habitat (Hertz, Huey & Stevenson, 1993). If lizards are unable to reach and maintain *T*_b_ within a range that maximizes the performance of functions directly related to fitness (i.e., locomotion, digestion, growth rate, reproduction), they will be forced to disperse to more suitable environments in order to survive (Vicenzi et al., 2017). Species with a high degree of endemicity, or which live in isolated patches and exhibit low vagility, might be at high risk of extinction (Chamaille-Jammes et al., 2006; Bonino et al., 2015b; Vicenzi et al., 2017). Furthermore, other studies propose that terrestrial ectotherms may have limited capacity to migrate in order to avoid negative effects on fitness due to global warming (Buckley, Tewksbury & Deutsch, 2013; Ofori et al., 2017).

The influence of temperature on physiological performance is generally studied by “thermal performance curves” (TPCs; Angilletta, 2009), which describe how a given performance trait responds to *T*_b_ (Huey & Stevenson, 1979; Huey & Bennett, 1987). The information provided by these curves, such as optimal temperature (*T*_o_), performance breadth, and critical temperatures, are essential to estimate the effects that changes in environmental temperature can induce in natural populations of ectotherms (Sinclair et al., 2016). In reptiles, one of the most used measurements of physiological performance is locomotor performance, because it reflects the interaction of several underlying physiological processes and is usually applied to ecological tasks relevant for natural and sexual selection, such as competition for food, predator avoidance, territory defense, and mate choice (Garland & Losos, 1994; Irschick & Garland, 2001; Irschick et al., 2008).

**Figure 1:**
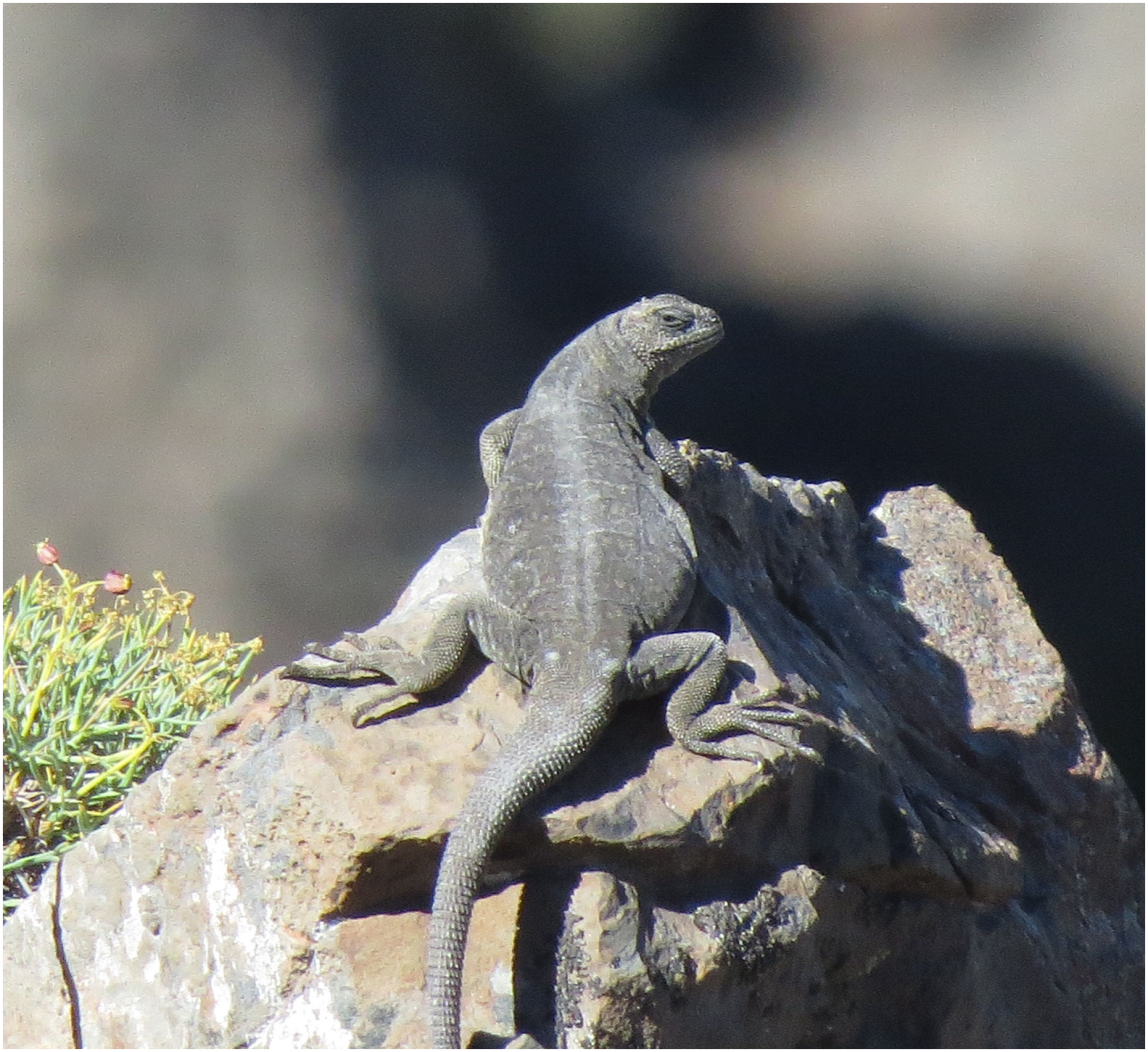
Adult of *Phymaturus tenebrosus*. Photo by Erika Kubisch.

Here, we study the thermal physiology and the thermal sensitivity of locomotor performance in the endangered viviparous lizard *Phymaturus tenebrosus* (Fig. 1). This species is a medium-sized lizard endemic to northwestern arid Patagonia (Perez et al., 2011). At present, *P. tenebrosus* is only known from a very restricted area in southwest Río Negro and Neuquén, Argentina (Kubisch et al., 2017). Recent climatic predictions for this region suggest an extreme increase in temperature of 3–3.5 °C and a decline in average daily precipitation of 0.75–1 mm by the end of this century, in comparison to current conditions (“representative concentration pathway” scenario 8.5, IPCC, 2014; Barros et al., 2015). Our focal species has been recently categorized by the IUCN as Endangered (Abdala, 2017), due to factors such as its limited and isolated distribution on rocky outcrops, specialization on a saxicolous and herbivorous lifestyle, viviparous reproductive mode, low annual reproductive output (Ibargüengoytía, 2004), and acquisition of sexual maturity at 7–9 years of age, with a maximum life span of 16 years (Piantoni, Cussac & Ibargüengoytía, 2006). In addition, Sinervo et al. (2010) predicts a high extinction risk for this lizard due to climate change. Other factors affecting its conservation are the high frequency of fires and sheep farming (Abdala et al., 2012), which intensify the natural fragmentation of its habitat.

In this paper, we hypothesize that: (1) the preferred body temperature of *P. tenebrosus* (*T*_pref_) is correlated with its *T*_o_ for locomotor performance, as postulated by the thermal coadaptation hypothesis (Huey & Bennett, 1987; Angilletta et al., 2006); (2) *P. tenebrosus* is capable of attaining high locomotor performance in the field by reaching and maintaining *T*_pref_ in the field, as has been observed in other species of the genus (Corbalán, Debandi & Kubisch, 2013; Gómez Alés, Acosta & Laspiur, 2017; Vicenzi et al., 2017; Duran, Kubisch & Boretto, 2018); (3) the potential increase in temperature due to global warming will affect locomotor performance of the species, considering the narrow thermal performance breadth observed in other *Phymaturus* (Bonino et al., 2015a; Gómez Alés et al., 2018; Vicenzi et al., 2019), increasing their vulnerability to extinction. Consequently, we predict that the *T*_o_ for locomotor performance will be included within the thermal preference range of the species, that *P. tenebrosus* is an efficient thermoregulator like other species of the genus, and that under a global warming scenario, locomotor performance will decrease, threatening the viability of the species.

## Materials and Methods

### Study site and field work

The study site is located on a rocky outcrop near Villa Llanquín, Río Negro, Argentina (40°52′S, 70°57′W, 1,200 m above sea level). Field work was carried out during summer (late January) of two consecutive years (2018 and 2019). This area is included within the Sub-Andean district of the Patagonic Phytogeographic Province (Cabrera, 1976), and it is characterized by a cool (mean annual air temperature: 10.66 °C) and semi-humid climate (mean total annual precipitation: 343 mm). The dominant landscape is barren steppe, with shrubby, low herbaceous coverage and bare soil percentages above 50% (Cabrera, 1978).

We captured 34 adult specimens of *P. tenebrosus* (15 females and 19 males) by hand or noose when they were active, between 900 to 1900 h. Considering the endangered conservation status of the species (Abdala, 2017) we aimed to work with the minimum sample size needed for statistical significance. Also, we did not include pregnant females in our sample, determining the reproductive status of females by palpation as in other *Phymaturus* (Boretto et al., 2014; Ibargüengoytía et al., 2016). Moreover, we used only a subset of individuals (*N* = 20) for the thermal tolerance experiments. Finally, our experimental design was planned to minimize the time spent in captivity and the stress suffered by the individuals.

Adult status was determined according to the minimum size at sexual maturity (85 mm in females and 87 mm in males) reported by Ibargüengoytía (2004). Immediately after capture, *T*_b_ was measured (TES 1,303, ± 0.03 °C digital thermometer) using a thermocouple (TES TP-K01, 1.62 mm diameter) inserted approximately 0.5 cm inside the cloaca. The temperature measurements were taken within 10 s of capture to prevent heat transfer from the operator's hands. We also recorded the substrate temperature (*T*_s_) and the air temperature one cm above the ground (*T*_a_) of the microenvironment where each lizard was captured.

Every capture site was georeferenced (three m resolution, GARMIN Map 60C Sx), and photographed (VC-03 Nikon camera, D3100) using a lizard model in the same position than the lizard was captured, allowing us to return each lizard to their exact capture site after experiments. A scientific collecting permit (Disposición No. 108/17) was obtained from the Secretaría de Ambiente y Desarrollo Sustentable of Rio Negro Province, Argentina. Lizards were cared for following the ASIH/HL/SSAR Guidelines for Use of Live Amphibians and Reptiles, as well as the regulations detailed in the Argentinean National Law N° 14346.

Lizards were carried to the laboratory in individual cloth bags. During experiments (4 days), lizards were kept in their respective individual bag at room temperature (20–22 °C) in a quiet site, taking care of the asepsis and avoided contact between individual lizards to minimize stress. After experiments, body weight (Pesola 50 ± 0.3 g) and snout-vent length (SVL; digital caliper Lee Tools ± 0.02 mm) were measured and sex recorded (based on the presence of pre-cloacal pores in males).

### Preferred body temperatures

Preferred body temperatures were measured the day after capture. Lizards were placed individually in an open-top terrarium (100 × 20 × 17 cm), with a thermal gradient (20–50 °C) produced by a 75 W incandescent light bulb in a lamp over one end of the terrarium. Lizard *T*_b_ were measured every 10 s for 2 h with a temperature Data Acquisition Module (USB-TC08; OMEGA, Biel/Bienne, Switzerland), using an ultra-thin (0.08 mm) catheter thermocouple, fastened to the belly and to the base of the lizard’s tail with hypoallergenic tape, to keep the thermocouple in position during the experiment. For each individual, we estimated the mean *T*_pref_ and the minimum and maximum temperature set-points (*T*_set_) as the central 50% of all *T*_b_ preferred in the laboratory.

### Effects of body temperature on locomotor performance

Locomotor performance trials were performed on a racetrack 0.075 m wide and 1.20 m long, leading to a shelter. Photocells, positioned at 0.15‐m intervals along the track, signaled passing lizards to a laptop that calculated speed over each 0.15‐m section. Two types of runs were considered in the analyses: (1) sprint runs (SR), defined as the speed reached between the first and the second photoreceptor (0.15‐m), which is relevant for predator escape and prey capture (Cabezas-Cartes, Kubisch & Ibargüengoytía, 2014), and (2) long runs (LR), defined as the speed between the first and the last photoreceptor (1.05 m), which indicated the locomotor capability of the lizard to perform activities such as foraging, territorial defense, and courtship (Cabezas-Cartes, Kubisch & Ibargüengoytía, 2014). Lizards ran three consecutive times at each experimental temperature, and only the maximum run speed (*V*_max_) for SR and LR of the three repetitions was considered for the analyses. Between these three consecutive trials lizards did not rest, but they were always rested at least 4 h between the different treatments.

Before and after each run, the lizard’s *T*_b_ was measured using a thermocouple inserted 0.5 cm inside the cloaca (catheter probe TES TP-K01) and connected to a digital thermometer (TES 1302; TES Electrical Electronic Corp., Taipei, Taiwan, China, ±0.01 °C). Trials were conducted at five *T*_b_ (20 ± 1 °C; 25 ± 1 °C; 30 ± 1 °C; 36 ± 1 °C; 39 ± 1 °C) during three consecutive days. The *T*_b_s were chosen based on the following criteria: the minimum temperature at which we found lizards active in the field (19 °C), the median *T*_b_ in the field (26.3 °C), and the mean *T*_pref_ (35.4 °C) obtained in the laboratory for *P. tenebrosus* (this study). The *T*_b_ at 30 °C was included to define the shape of the curve. Also, we estimated performance at 39 °C, to evaluate the locomotor performance at the mean *T*_pref_ + 3.5 °C, simulating a potential shift in field active *T*_b_ (Gilbert & Miles, 2017) anticipated by the end of this century at our study site, given the climatic assessment from the IPCC (2014; Barros et al., 2015). Locomotor performance trials were carried out in an environmental chamber equipped with an air conditioner, heat lamps, electric heaters, and a thermostat to set the desired temperature. Lizards were placed at the specified temperature 1 h prior to the run, following the methods of Angilletta, Hill & Robson (2002), Fernández et al. (2011), Kubisch, Fernández & Ibargüengoytía (2011), and Kubisch et al. (2016).

### Thermal tolerance

To determine the critical thermal minimum (CT_min_), a subsample of 20 lizards were placed individually in a plastic transparent box (15 × 10 × 5 cm) in a refrigerator at −10 °C. *T*_b_ was measured every 15 s (with a temperature Data Acquisition Module, USB-TC08; OMEGA, Biel/Bienne, Switzerland) using an ultra-thin (0.08 mm) catheter thermocouple fastened to the belly and to the base of the lizard’s tail. Lizards were observed throughout the experiment and we recorded and considered as CT_min_ the *T*_b_ at which an individual was no longer able to right itself when placed on its back.

The same subsample of 20 lizards were also used to determine critical thermal maximum (CT_max_). Each lizard was placed in an open-top terrarium (15 × 20 × 20 cm) with an infrared 150-W lamp 40 cm overhead. The *T*_b_ was monitored every 15 s following the same methodology used to the CT_min_. Each lizard was carefully observed throughout the experiment and we recorded and considered as CT_max_ the *T*_b_ at which an individual was unable to right itself when it was placed on its back. After reaching CT_max_, each lizard was removed from heat and cooled quickly to avoid overheating.

### Operative temperatures, effectiveness of thermoregulation, and vulnerability to global warming

The operative temperature (*T*_e_) represents the “null” distribution of *T*_b_ that non-thermoregulating animals would achieve in their environment (Hertz, Huey & Stevenson, 1993). The *T*_e_ was measured using gray PVC oval plastic models 35 × 10 mm in cross-section and 120 mm long, to mimic an adult *P. tenebrosus*. The model was chosen to represent a living animal and was validated for a *Phymaturus* species of similar shape and size to *P. tenebrosus*, showing a strong association between the model temperature and the live animal temperature (*R* = 0.98; Gómez Alés, Acosta & Laspiur, 2017). Each model was connected to a thermistor and both ends were sealed with silicone (Fastix^®^, AKAPOL S.A., Buenos Aires, Argentina). The thermistor was connected to data loggers (HOBO Onset Computer Corporation, Bourne (MA), USA). The models were placed in the most extreme thermal situations present at our field site: three models inside crevices (shelters used by lizards) and three models exposed on the rock. *Phymaturus* lizards usually shift between sun patches and rock crevices to thermoregulate (Duran, Kubisch & Boretto, 2018). The data loggers were programed to record temperatures every 30 min during 2 months of the lizards’ active season (from the end of January to the end of March). In our analyses, we used the *T*_e_s recorded between 900 and 1,900 h (daily period of activity; personal observation).

In order to estimate the thermoregulatory effectiveness of a subset of our sample of *P. tenebrosus* (*N* = 11), we applied the methodology of Hertz, Huey & Stevenson (1993), using the information on *T*_pref_ obtained in the thermal gradient and the availability of *T*_e_ in the natural environment. We defined the *d*_b_ (individual deviation) as an index based on the average extent to which each individual experienced *T*_b_ outside the set-point range of their *T*_pref_. The *d*_b_ was estimated as the average of the absolute value of the deviations of *T*_b_ from *T*_set_ of each individual. Then, we calculated the *d*_e_ as an index of the mean thermal quality of the habitat from an organism’s perspective. The *d*_e_ was estimated as the mean of the deviation of *T*_e_ from the *T*_set_ of each individual. Considering the high daily variation in temperature that characterizes the Patagonian climate, the *T*_e_ is highly dynamic. Hence, we used the mean value of *T*_e_ from the overall models recorded from one hour before each individual lizard was captured (Duran, Kubisch & Boretto, 2018). The effectiveness of thermoregulation (*E*) was defined as the degree of active selection of the thermal microhabitat and calculated using the formula *E* = 1 − (*d*_b_/*d*_e_), which integrates the average degree to which *P. tenebrosus* experienced *T*_b_ outside the set-point range (*d*_b_), and the corresponding *d*_e_. When the *E* index approaches zero the species is considered to be a thermoconformer, whereas when the *E* index approaches a value of one the species can be considered a highly effective thermoregulator. Finally, if the *E* index is close to 0.5, the species is considered to be a moderate thermoregulator (Hertz, Huey & Stevenson, 1993).

In addition, we calculate two indices to estimate vulnerability to global warming: the warming tolerance (WT), which defines how much warming can be tolerated by an ectotherm before its performance is reduced to lethal levels, and is defined as the difference between mean CT_max_ and mean *T*_e_ (sensu Deutsch et al. (2008) and Logan et al. (2013)). Also, we determined the thermal safety margin (TSM) for physiological performance as the difference between the *T*_o_ and mean *T*_e_, which gives an indication of how close animals’ thermal optima are to the current climatic temperature in their environment (Deutsch et al., 2008; Andrew et al., 2013).

### Statistical analyses

To quantify the thermal sensitivity for locomotor performance, we used Table Curve 2D v5.01.2 software to create locomotor performance curves (TPCs) as function of temperature during SR and LR. The values of CT_min_ and CT_max_ were used as the extreme values of the curves (speed equal to 0 m/s). We chose the model according to the adjusted *R*^2^ and the lowest Akaike information criterion (AIC) (following Angilletta (2006)). Also, we estimated the differential AICc (Δ*i*), which is the difference between a given model’s AIC and the lowest AIC, and the Akaike weight (w_i_), as a measure of the strength of the evidence for each model, indicating the probability that a given model is the best among a series of candidate models (Burnham & Anderson, 2002). The best candidate model for the overall sample was fitted to each individual in order to estimate the *V*_max_, the *T*_o_ (the *T*_b_ at which performance is maximal) and the performance breadth (*B*_80_, the range of *T*_b_s over which performance is greater than or equal to 80% of the *V*_max_; sensu Ben-Ezra, Bulté & Blouin-Demers (2008)) of each individual for LR and SR.

We used the statistical software programs Sigma Stat 3.5^®^ and Sigma Plot 14.0^®^ for statistical analyses and to generate figures. Relationships between variables were analyzed by simple linear regressions. To compare two related samples, we used paired *t*-tests or the non-parametric alternative Wilcoxon signed rank test when the assumptions of parametric statistics were not met. Means are reported ± standard error.

## Results

### Body measurements and their relationship with locomotor performance

The mean SVL of *P. tenebrosus* (*N* = 34) at Villa Llanquín was 92.19 (± 0.85 mm) and the median body mass was 27.65 (range 14.12–35.00 g). There was no relationship between maximum speeds during SR or LR and SVL (linear regression: *F*_SR 1,29_ = 0.40; *F*_LR 1,31_ = 0.005; *P* > 0.5) or body mass (linear regression: *F*_SR 1, 28_ = 1.41; *F*_LR 1, 30_ = 0.18; *P* > 0.5).

### Field temperatures

**Figure 2:**
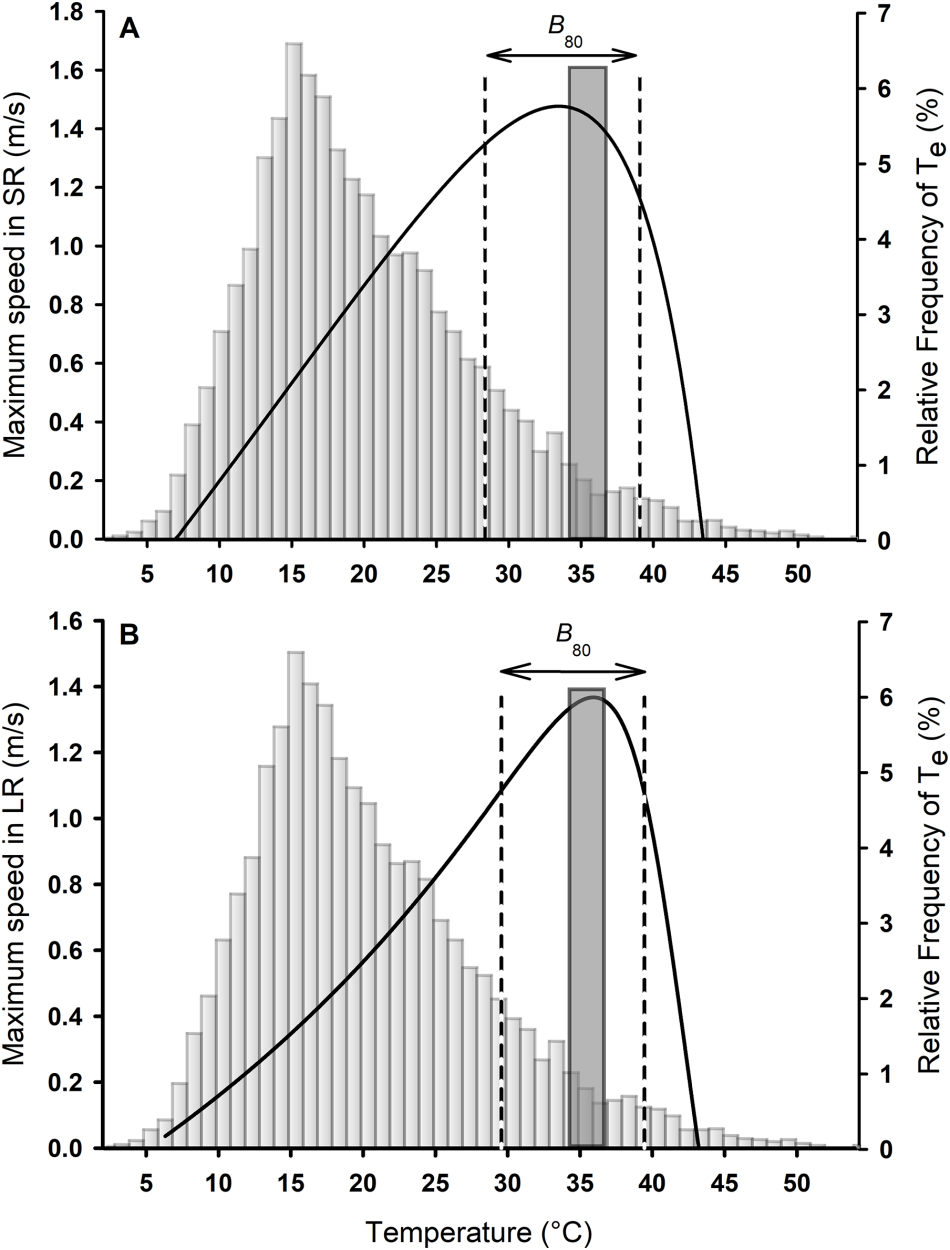
Thermal performance curves for sprints (A) and long runs (B) for *Phymaturus tenebrosus*. Vertical dashed black lines represent performance breadth during SR and LR (*B*_80_). Gray bars represent the percentage frequency distribution of all operative temperatures between activity hours, from January to March 2019. The dark gray bar and striped bar represent the set point range of *T*_pref_ obtained in the laboratory.

**Figure 3:**
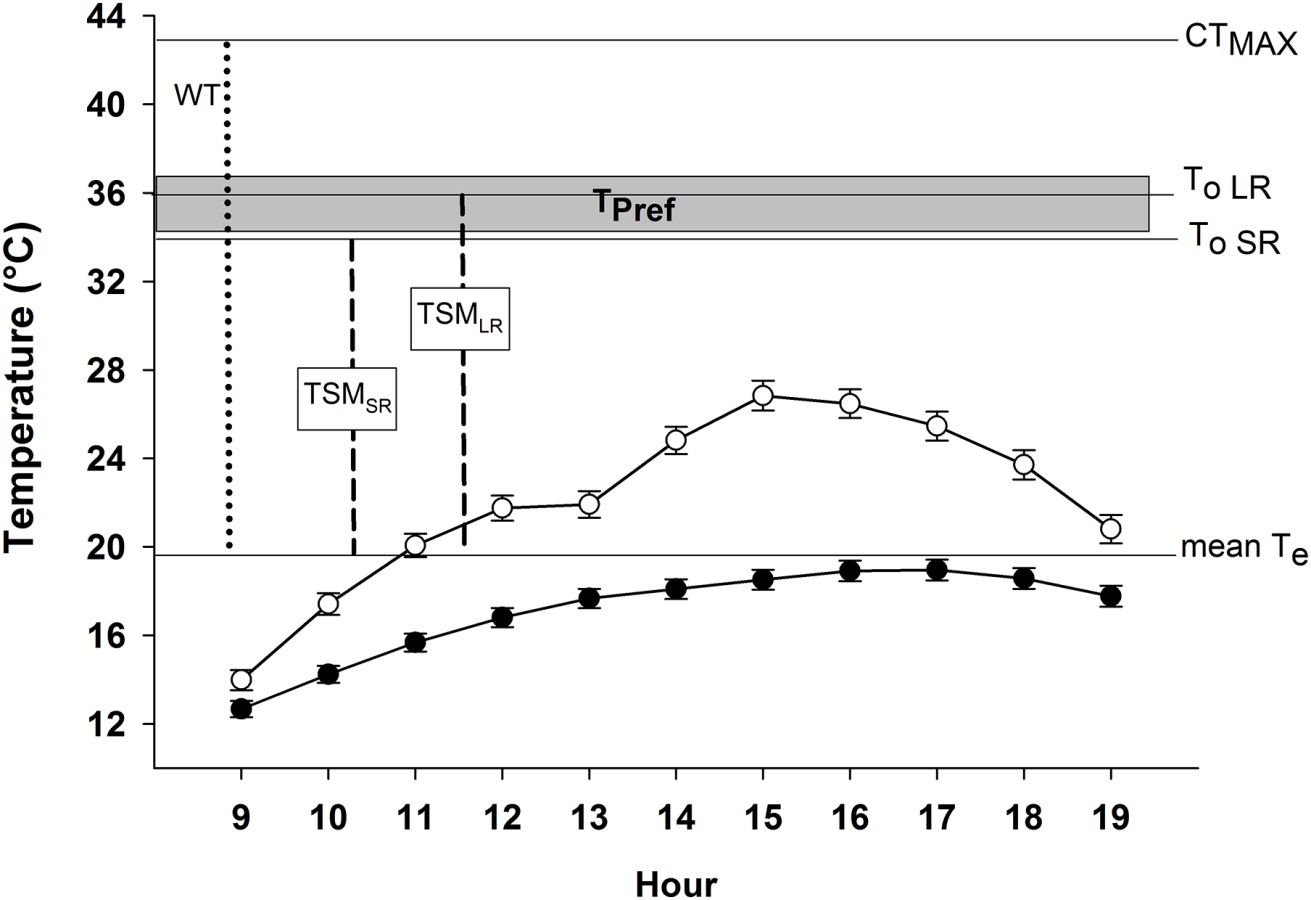
Microenvironmental temperatures (*T*_e_) of exposed models (empty circles) and models inside crevices (filled circles) during activity period of *Phymaturus tenebrosus* in Villa Llanquín. The critical thermal maximum (CT_Max_), optimal temperature during SR (*T*_o SR_), and LR (*T*_o LR_), are indicated with horizontal lines, as well as the mean *T*_e_. In addition, the warming tolerance (WT, dotted line) and thermal safety margin (TSM, dashed lines) indexes are shown, as well as the set point range of the preferred temperature (*T*_pref_, gray rectangle).

The median *T*_b_ in the field was 26.3 °C (range 16.50–33.00 °C). Lizards were captured in microenvironments with a mean *T*_a_ of 23.08 ± 0.67 °C, and a mean *T*_s_ of 23.85 ± 0.88 °C. The mean *T*_e_ for all physical models was 19.59 ± 0.09 °C (range = 1.15–53.74 °C; Figs. 2 and 3). The mean *T*_e_ of exposed models was 22.10 ± 0.21 °C, while the mean *T*_e_ of models placed inside crevices was 17.07 ± 0.14 °C.

### Thermal performance curves

The relationship between *T*_b_ and maximum speed for the overall sample was best described by a curve of the Pearson family for both SR (Adj *R*^2^ = 0.57; Fig. 2A) and LR (Adj *R*^2^ = 0.85; Fig. 2B) according to the lowest AIC. Lizards ran faster during SR than LR (Median *V*_max_ SR = 1.65 m/s, *V*_max_ LR = 1.38 m/s; Wilcoxon signed rank test: *W* = −357.000; *P* < 0.001; Fig. 2). However, the *T*_o_ was similar between SR and LR (Median *T*_o_ SR = 35.18 °C, *T*_o_ LR = 36.09 °C; Wilcoxon signed rank test: *W* = 182.000; *P* = 0.08; Fig. 2). Considering the thermal performance breadth at which 80% of maximal speed could be reached (*B*_80_) there was no difference between SR and LR (*B*_80 SR_ = 28.36–39.07, *B*_80 LR_ = 29.57–39.46; Paired *t*-test: *t*_30_ = −1.29, *P* = 0.21, Fig. 2). The mean *B*_80_ for SR was 8.95 ± 0.71 °C, while the mean *B*_80_ for LR was 9.93 ± 0.41 °C.

### Thermal physiology of *P. tenebrosus*, effectiveness of thermoregulation, and vulnerability to global warming

The mean *T*_pref_ of *P. tenebrosus* in the laboratory was 35.36 ± 0.38 °C with a set-point of *T*_pref_ of 34.09–36.59 °C (Figs. 2 and 3). The *T*_pref_ was similar to the mean *T*_o_ for both SR and LR (SR: Wilcoxon signed rank test: *W* = 36.000; *P* = 0.70, Fig. 2A; LR: Paired *t*-test: *t*_30_ = 0.90; *P* = 0.37, Fig. 2B). The mean critical minimum and maximum temperatures were CT_min_ = 7.62 ± 0.20 °C and CT_max_ = 42.90 ± 0.21 °C, respectively.

Our results indicate that *P. tenebrosus* has a very low effectiveness of thermoregulation (*E* = 0.19), primarily because it exhibits a low accuracy of thermoregulation (*d*_b_ = 9.28), and inhabits a low-quality thermal environment (*d*_e_ = 11.48). The indices to estimate its vulnerability to global warming were: WT = 23.30 °C and TSM for SR = 15.58 °C and for LR = 16.30 °C (Fig. 3).

## Discussion

The interplay between environmental temperature and thermal physiology in ectotherms can be analyzed by several physiological parameters (such as *T*_pref_, TPCs, CT_max_, CT_min_, WT, and TSM), which are useful to predict the ecological consequences of climate warming for individual fitness and, therefore, to assess the vulnerability of ectotherms to climate change (Deutsch et al., 2008; Sinclair et al., 2016; Gilbert & Miles, 2017). As we predicted, *P. tenebrosus* reached their *T*_o_ in both types of runs at temperatures within the thermal preference range (during LR) or near the lower *T*_set_ of *T*_pref_ (during SR). The *T*_o_ was similar to what was previously reported for a small sample of this species (*T*_o_ = 35.4 °C, *N* = 3; Bonino et al., 2015a). There are only two other species in the genus *Phymaturus* for which TPCs have been published. In the case of *P. extrilidus* the *T_o_* (LR = 32.5, SR = 33.25) was lower than their *T*_pref_ (35.74), but they attained their maximum locomotor performance at temperatures they generally experienced during daily activity (Gómez Alés et al., 2018). In contrast, *P. palluma*, similarly to *P. tenebrosus*, exhibited *T*_pref_ that optimized their performance (*T*_o LR_ = 35.9, *T*_o SR_ = 35.18, *T*_pref_ = 35.36; Vicenzi et al., 2019). These results reinforce the hypothesis of coadaptation of thermoregulatory behavior and thermal physiology, which states that individuals select *T*_b_ that optimize their physiological functions and, hence, fitness (Huey & Stevenson, 1979; Huey & Bennett, 1987; Angilletta, Hill & Robson, 2002; Bonino et al., 2011).

The *T*_e_ and field *T*_b_ for *P. tenebrosus* are below its *T*_pref_ and also below the temperatures at which these lizards reach maximum locomotor performance (*T*_o_ and *B*_80_, for SR and LR). In addition, the indices of efficiency of thermoregulation showed that *P. tenebrosus* has the lowest *E* index reported for the genus (Corbalán, Debandi & Kubisch, 2013; Gómez Alés, Acosta & Laspiur, 2017; Vicenzi et al., 2017; Duran, Kubisch & Boretto, 2018), with limited ability to achieve *T*_pref_ in their natural environment. This lizard can thus be considered a poor thermoregulator, similar to *Liolaemus sarmientoi* from southern Patagonia (50°S; *E* = 0.30, mean *T*_b_ = 26.18 °C; Ibargüengoytía et al., 2010). Despite of living in cold-temperate climates, *P. tenebrosus* as several other Liolaemidae lizards, presents a conservative high value of *T*_pref_, generally higher than the mean *T*_e_ and mean *T*_b_ (Cruz et al., 2009; Labra, Pienaar & Hansen, 2009; Rodríguez-Serrano, Navas & Bozinovic, 2009; Ibargüengoytía et al., 2010; Kubisch, Fernández & Ibargüengoytía, 2011; Medina et al., 2012; Moreno Azócar et al., 2013; Corbalán, Debandi & Kubisch, 2013). This could be partially explained by the fact that thermoregulation is an expensive behavior that increases energy expenditures and risk of predation (Sears & Angilletta, 2015) and also by the low availability of *T*_pref_ in the microenvironments that most of Patagonian lizards inhabit.

*Phymaturus tenebrosus* lives under harsher conditions than other *Phymaturus* from Patagonia, because it lives at high latitudes and also next to the Andes, which produces a colder and more humid climate (Kubisch et al., 2017), than the steppe habitats where other congeners occur (Cabezas-Cartes, 2016). For example, the Patagonian *P. querque* (*E* = 0.44) and *P. zapalensis* (*E* = 0.47) from Zapala, Neuquén (39°S, 70°W) experience higher *T*_e_ than their mean *T*_b_ and *T*_pref_ (Duran, Kubisch & Boretto, 2018), which allow them to thermoregulate more effectively than *P. tenebrosus*. Whereas northern species of the genus like *P. payuniae* (*E* = 0.64; Corbalán, Debandi & Kubisch, 2013), *P. palluma* (*E* = 0.79; Vicenzi et al., 2017), and *P. extrilidus* (*E* = 0.65; Gómez Alés, Acosta & Laspiur, 2017) are also more efficient thermoregulators than *P. tenebrosus*, despite of inhabiting low thermal quality environments.

Most recent studies in lepidosaurians have found negative effects of climate change, highlighting these animals’ vulnerability to temperature variation (Diele-Viegas & Rocha, 2018). However, it was also suggested that thermal tolerance breadths of ectotherms generally increase with latitude, and also that these ectotherms live at cooler temperatures than their physiological optima, so warming may enhance fitness in high-latitude species (Deutsch et al., 2008; Fernández et al., 2011; Sunday et al., 2014; Bonino et al., 2015a). In the case of *P. palluma* from the highlands of the Central Andes (Vicenzi et al., 2019) the *T*_pref_ and *T*_o_ are very similar to those found for *P. tenebrosus* in this study, but the indices to estimate vulnerability to global warming are substantially different. *P. palluma* experiences *T*_e_ that included the *B*_80_ and the set point range of *T*_pref_, but has very low values of WT and TSM. Thus, the authors estimated that with an increase in temperatures due to global warming, this Andean species will become vulnerable to extinction. In the case of *P. tenebrosus*, in contrast to our predictions, the indices to estimate vulnerability to global warming are very high, indicating that lizards of this species cannot reach temperatures near their *T*_o_ for locomotor performance or their *T*_pref_. So, as was also suggested for the congener *P. extrilidus* (Gómez Alés et al., 2018), the southernmost reptiles of the world (*L. sarmientoi* and *L. magellanicus*, Fernández et al., 2011), and other liolaemids (Bonino et al., 2015a), our study species has a wide safety margin until fitness would decline, and may actually benefit from predicted climate warming for at least the next few decades.

Studies on thermal physiology in lizards generally reveal that climate change will allow them fewer hours of activity, with few opportunities to reproduce and forage, and will reduce performance capacity (Sinervo et al., 2010; Buckley, Tewksbury & Deutsch, 2013; Gilbert & Miles, 2017). For example, the Patagonian congeners *P. querque* and *P. zapalensis*, as well as the Andean *P. palluma*, are forced to retreat to cool refuges to avoid overheating during their active period because of high *T*_e_s (Vicenzi et al., 2017; Duran, Kubisch & Boretto, 2018). *P. tenebrosus* is an endangered species with ecological conditions that increase their vulnerability to global warming. However, the ability to withstand the impact of habitat warming might be important for this species to survive future global change. The present study together with some other studies on thermal physiology and locomotor performance of Andean and Patagonian lizards (Bonino et al., 2011, 2015a; Fernández et al., 2011; Gómez Alés et al., 2018), suggest these animals are not likely to be threatened by the higher temperatures predicted as a result of global warming.

## Conclusions

*Phymaturus tenebrosus* exhibit its maximum locomotor performance at temperatures similar to its *T*_pref_. These temperatures are substantially higher than the mean temperature it currently experiences in its habitat. In addition, it exhibits a low effectiveness of thermoregulation, being a poor thermoregulator. Further research should analyze if current *T*_e_ (similar to the mean *T*_b_) experienced by *P. tenebrosus* are optimal temperatures for other biological processes such as bite force, stamina, feeding rate, growth rate, or behavioral traits (territorial defense, courtship). However, in view of the results obtained, specifically based on the high TSMs estimated, we suggest that considering the higher temperatures predicted as a result of global warming, *P. tenebrosus* is likely to experience climatic conditions in Patagonia that may enhance its thermal physiology and locomotor performance, favoring the survival of this species to future global climate change.

## Supplemental Information

10.7717/peerj.7437/supp-1 Supplemental Information 1 Raw data for microenvironmental temperatures in Fig. 3. Maximum speed in sprint and long runs at the different temperature treatments.

10.7717/peerj.7437/supp-2 Supplemental Information 2 Raw data of operative temperatures, preferred temperatures, and optimal temperatures of *P. tenebrosus*.

## Additional Information and Declarations

### Field Study Permissions

The following information was supplied relating to field study approvals (i.e., approving body and any reference numbers): A scientific collecting permit (Disposición No. 108/17) was obtained from the Secretaría de Ambiente y Desarrollo Sustentable of Rio Negro Province, Argentina.

### Data Availability

The following information was supplied regarding data availability: The raw data are available in the Supplemental Files. The raw data shows all maximum speed achieved in sprint and long runs in each temperature trial by each lizard.
